# Detecting Airway Invasion in Variable-Length Videofluoroscopic Swallowing Studies: A Vision Transformer Approach for Oropharyngeal Dysphagia

**DOI:** 10.3390/diagnostics15243180

**Published:** 2025-12-12

**Authors:** Hesam Abdolmotalleby, Joseph M. Reinhardt, Douglas J. Van Daele

**Affiliations:** 1 Roy J. Carver Department of Biomedical Engineering, University of Iowa, Iowa City, IA 52242, USA; joe-reinhardt@uiowa.edu; 2 Department of Otolaryngology, University of Iowa, Iowa City, IA 52242, USA; douglas-van-daele@uiowa.edu

**Keywords:** dysphagia, videofluoroscopy (VFSS), deep learning, vision transformer (ViT)

## Abstract

**Background:** Dysphagia from aging, neurodegeneration, structural anomalies, or cognitive decline harms quality of life. The videofluoroscopic swallowing study (VFSS) is the diagnostic gold standard but manual interpretation is labor-intensive and costly, motivating automation. **Methods:** We introduce a Vision Transformer (ViT) using a temporal sliding window and 3D patch tokenization to capture spatio-temporal dependencies in variable-length VFSS via attention. Training/evaluation used 1154 VFSS sequences from 107 individuals (548 abnormal, 606 normal) with 5-fold cross-validation and comparisons to VGG-16, ResNet-50, EfficientNet-V1/V2, and MobileNet. **Results:** The ViT achieved 84.37 ± 1.15% accuracy, 90.81 ± 2.11% sensitivity, 79.49 ± 1.66% specificity, 82.94 ± 2.76% precision, 85.68 ± 1.54% F1-score, and AUC 0.878 (5-fold). It outperformed all CNN baselines across metrics; paired *t*-tests confirmed significant gains (*p* < 0.05). **Conclusions:** The pure ViT’s attention-based spatio-temporal modeling yields robust VFSS classification and is well-suited for screening workflows requiring timely abnormality detection, providing a foundation for clinically deployable VFSS analysis.

## 1. Introduction

Dysphagia, defined by difficulties in transferring food or liquid boluses from the mouth to the stomach, can develop under various circumstances. These include advanced age, stroke or Parkinson’s disease among other neurological conditions, structural anomalies such as cancer or fistulae, and cognitive decline that may occur in disorders like dementia [1]. Dysphagia affects roughly 16–23% of individuals in the general population [2]. This condition can lead to significant adverse effects such as social isolation, diminished quality of life, and heightened risks of aspiration, pneumonia, choking, malnutrition, and dehydration [3,4].

The videofluoroscopic swallowing study (VFSS) is considered the gold standard for the diagnosis and monitoring of dysphagia. VFSS involves recording video images using a fluoroscope while patients swallow foods of different viscosities, allowing physicians to observe the trajectories and kinematics of the bolus from the oral phase to the stomach [5,6,7]. These video recordings are subsequently analyzed by experts for diagnostic purposes. Each subject requires multiple recordings for each food consistency, which must be assessed frame-by-frame. Consequently, this process is labor-intensive, necessitating specialized expertise, thereby increasing the cost and reducing the availability of the examination [8].

Numerous methodologies have been proposed to automate the VFSS interpretation process. Initial approaches incorporated signal processing techniques with minimal expert annotation [9,10,11]. With the advent and advancement of deep learning techniques in recent years, novel methods have sought to exploit the computational efficiency and reliability of these approaches while minimizing user input for classification tasks. A number of studies have pursued automated airway invasion detection using both image- and video-based approaches, yet many require prior data manipulation, such as frame preselection or cropping, which limits their clinical applicability. For instance, several image-based methods extract features from manually identified frames: Lee et al. [12] employed a Xception-based CNN achieving 97.2% frame-level accuracy and 93.2% video-level accuracy, while Kim et al. [8] used a U-Net architecture to classify scans based on a coarse penetration-aspiration scale, reporting an average area-under-the-curve (AUC) of up to 0.961. Similarly, another approach by Kim et al. [13] used MobileNet to analyze five consecutive frames around the highest and lowest hyoid positions, achieving an AUC of 0.942. In contrast, Iida et al. focused on preselected pharyngeal regions and employed a Single-Layer CNN, resulting in an AUC of 0.974. Video-based techniques, such as the CNN-LSTM method by Reddy et al. [14]—which used either a set of 10 or 25 frames to capture temporal dependencies—have also been explored, achieving an accuracy of 87.92%. However, all these methods share a critical limitation: the necessity for pre-processing or altering the data (e.g., through manual frame selection) before network input in inference phase, rendering them less suitable for real-time clinical use.

Recent advances in vision transformers have substantially reshaped medical image and video analysis, where self-attention enables modeling of long-range spatial and temporal dependencies that are difficult to capture with purely convolutional architectures. In gastrointestinal endoscopy, for example, endoscopy-pretrained Vision Transformer models such as EndoViT, trained on hundreds of thousands of minimally invasive surgery images, and transformer encoders that process entire capsule endoscopy videos rather than isolated frames, have demonstrated strong performance across lesion detection and other downstream tasks by exploiting global spatio-temporal context [15,16]. In cardiac imaging, hybrid CNN-transformer models like CardSegNet and deformation-encoding deep learning transformers for cine MRI leverage attention to jointly model local texture and global motion, improving heart region segmentation and enabling high-frame-rate cine reconstruction without sacrificing spatial resolution [17,18]. Similarly, in surgical video analysis, vision transformer architectures and specialized video transformers such as the SKiT model have been shown to accurately decode surgeon activity and recognize surgical phases in long laparoscopic sequences by attending over extended temporal windows [19,20]. By contrast, most prior work on automated analysis of VFSS has relied on convolutional networks or encoder-decoder architectures, including ResNet3D-based models for temporal parameter estimation, 3D CNN action-recognition networks for laryngeal invasion detection, and CNN or U-Net variants for bolus segmentation, often operating on carefully trimmed clips or frame-level annotations rather than full examinations [21,22,23]. Only a small number of recent VFSS studies have incorporated transformer components, primarily for segmentation tasks: Video-SwinUNet combines CNNs with Swin transformer blocks to segment bolus and airway-related structures from short VFSS sequences, while temporally blended ViT backbones such as Video-TransUNet have been explored for CT-VFSS segmentation under dense pixel-wise supervision [24,25].

Building on this emerging literature, our study introduces a ViT-based algorithm that explicitly leverages a transformer-based architecture for VFSS classification in a weakly-supervised regime, operating directly on full-length clinical studies with only study-level labels and no manual frame selection or detailed frame/event annotations. By employing a spatio-temporal sliding window, the model classifies entire video clips as normal or abnormal without any preparatory modifications, thereby exploiting transformers’ capacity to model long-range spatio-temporal dependencies across entire swallowing examinations and distinguishing our approach from the predominantly CNN-based or segmentation-focused transformer methods used previously in VFSS. To the best of our knowledge, this is the first transformer model implemented for the task of VFSS classification, and it is particularly well-suited for clinical scenarios where a large corpus of data is available for training. Unlike other models reported in the literature, the proposed framework is fully automated, requiring no human intervention after training and operating independently. Rather than serving as a direct replacement for routine clinical judgment, this framework is best viewed as a proof-of-concept that demonstrates the feasibility of fully automated transformer-based VFSS classification and, with further validation and prospective evaluation, could be integrated as a complementary decision-support tool to assist clinicians in dysphagia assessment and ultimately contribute to improved patient care.

## 2. Dataset

The dataset comprised 1154 retrospectively gathered VFSS clips from 107 individuals, including 548 abnormal and 606 normal swallowing events, as determined by a speech-language pathologist (SLP). Recordings were captured with one of four devices—Kodak CR950, NAI Tech Products MDR Video, PACSGEAR MDR Video, or Siemens Fluorospot Compact—all of which digitize the continuous analog output of the radiographic fluoroscopy process. In most cases, videos were taken at 25 frames per second, yielding an average of 103 frames per study (approximately 4 s per clip) at a resolution of 1015×1015 pixels per frame. Clips shorter than 25 frames were excluded from the analysis, as a typical swallow event is expected to span approximately 1 s or more; sequences below this threshold are therefore unlikely to contain a complete swallow and do not provide sufficient temporal context for reliable model training or evaluation. The distribution of all videos across scanners is summarized in Table 1.

## 3. Dataset Preprocessing

Building on the methodology established by Wilhelm et al. [26], the VFSS preprocessing pipeline applies a uniform, fully automated sequence of steps to both training and testing data. Unlike methods requiring manual frame selection or other forms of human oversight, this pipeline operates automatically, facilitating its use in clinical environments without additional supervision. Initially, VFSS files are labeled as “normal” or “abnormal” by an SLP, and files without these labels are discarded. Key information such as sequence length, frame resolution, and pixel data are extracted to determine if the VFSS represents a swallowing event. Sequences with fewer than 25 frames or with narrow pixel resolution are excluded, as they likely do not represent valid swallowing sequences.

Valid VFSS clips are treated as a series of image frames. The recordings, initially captured in RGB channels, consist of two duplicate channels of the first. Consequently, only the first channel, representing the image brightness, is retained to enhance processing efficiency. Frames initially contain a patient information window, which is cropped using a horizontal Prewitt operator. Early frames often contain a targeting artifact used for alignment and contrast adjustment, which is extracted using a Hough transform and inpainted based on a predefined mask.

**Figure 1:**
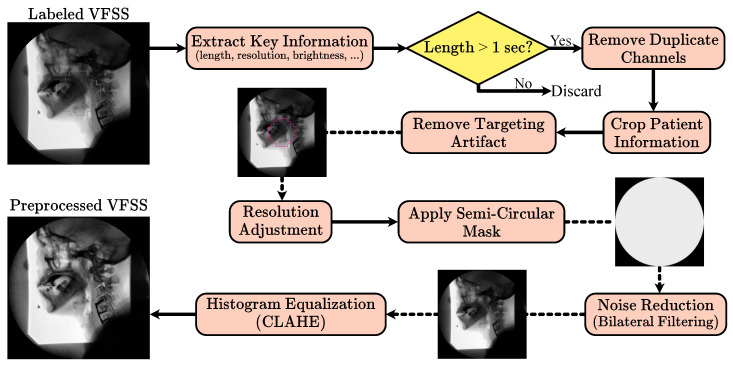
Overview of the VFSS preprocessing pipeline, including frame extraction, adjustment, artifact removal, denoising, and histogram equalization.

VFSS clips below the median resolution are zero-padded, while those above are cropped to maintain uniform dimensions. A mask is applied to ensure that the semi-circular features at the detector window’s corners maintain a uniform zero value. Subsequently, a bilateral filter reduces noise, and contrast-limited adaptive histogram equalization (CLAHE) enhances local structure contrast by equalizing smaller image tiles while curbing noise amplification. The corner mask is reapplied after CLAHE to further reduce noise artifacts. Figure 1 shows the preprocessing pipeline.

To achieve a uniform preprocessing strategy, we empirically tuned and then fixed all preprocessing parameters across scanners and studies. The CLAHE clip limit and tile grid size were selected through an empirical grid search with visual and histogram-based inspection of a stratified subset of examinations from each fluoroscopy system, ensuring that contrast in key regions such as the oropharynx and laryngeal vestibule was consistently enhanced without amplifying noise or obscuring anatomical detail. The circular field-of-view mask is defined to exclude peripheral border regions that contain mainly noise or non-anatomical content; this mask is applied before denoising and reapplied after CLAHE so that pixels outside the active detector area are consistently set to zero. Cropping is performed deterministically, with margins derived from the original acquisition layout so that static overlays and text bands containing patient identifiers or institutional labels are removed while the internal fluoroscopic field containing the patient and bolus is fully preserved, thereby de-identifying the data without changing the clinical field of view. In the subsequent 3D patch extraction step, we enforce a fixed temporal patch length of 25 frames by applying zero padding only at the end of videos whose length is not an exact multiple of this window, which yields patches of uniform temporal size without discarding any observed. Together, these design choices result in a fully automated preprocessing pipeline whose parameters are quantitatively justified and applied consistently across all studies and devices.

## 4. Method

This study used routinely acquired VFSS examinations to train and test an automated deep learning model that classifies entire studies as normal or abnormal with respect to swallow safety. For each patient, the full fluoroscopic video sequence was collected and preprocessed in a standardized way, then provided to a computer-based pattern recognition system that learns image and motion patterns associated with normal versus abnormal swallowing without any manual frame selection or region of interest annotation by human experts. Methodologically, this corresponds to a binary video classification task that we address using a vision transformer-based architecture adapted to handle 3D patches extracted from the single channel VFSS videos. Unlike standard 2D patch-based vision transformers that model spatial information frame by frame, our approach operates on small spatio-temporal patches that jointly encode spatial appearance and temporal evolution, which are then fed into a transformer encoder to learn a rich representation of the swallowing sequence. The encoder output is finally mapped to a single probability reflecting the likelihood that the study is abnormal, and the model is optimized using a binary cross-entropy loss while performance is evaluated on independent test data by comparing model predictions with clinician assigned labels using standard diagnostic accuracy metrics. In the following, we discuss the method in detail.

**Input Data and Preprocessing:** We assume a single-channel video *V* of dimension (T,H,W,1), where *T* is the number of frames, and H×W is the spatial resolution of each frame. Before tokenization, we apply intensity normalization (e.g., scaling pixel values to [0,1]) This step ensures stable training and helps the model generalize better.

**3D Patch Extraction:** Instead of processing frames individually, we segment the entire video into a grid of 3D patches. Each patch spans $T_{p}$ frames in time and covers a P×P spatial region. Formally, we partition the video along the temporal dimension into $\frac{T}{T_{p}}$ segments, and each segment along the height and width into $\frac{H}{P}$ and $\frac{W}{P}$ blocks, respectively. Thus, the total number of 3D patches is $N=\frac{T}{T_{p}}\times \frac{H}{P}\times \frac{W}{P}$. Each 3D patch $x_{i}\in R^{T_{p}\times P\times P\times 1}$ is then flattened into a vector $x_{i}\in R^{T_{p}\cdot P^{2}}$. For simplicity, we assume that *T*, *H*, and *W* are divisible by $T_{p}$ and *P*. If this is not the case, patches could be extracted by cropping the video to a divisible size or using padding. The choice of $T_{p}$ and *P* controls the granularity of the spatio-temporal information captured by each token. Smaller patches capture finer details at a higher number of tokens, while larger patches reduce the sequence length but may lose fine-grained information.

**Patch Embedding:** To transform raw 3D patches into a feature representation suitable for the transformer, we apply a learnable linear projection $z_{i}^{\left(0\right)}=Ex_{i},\;z_{i}^{\left(0\right)}\in R^{D}$, where $E\in R^{(T_{p}\cdot P^{2})\times D}$ is a weight matrix. “(0)” marks the “zero-th” stage or initial embeddings before the transformer operations begin. This convention is used to distinguish the initial state (0) from subsequent states (1),(2),…,(L) after passing through multiple layers of the transformer. This step maps each flattened patch into a *D*-dimensional latent space. The dimension *D* is a critical hyperparameter: larger *D* typically allows the model to capture more complex representations but increases computational cost. We initialize E using standard initialization schemes and optimize it jointly with the rest of the model parameters. After projection, we obtain a sequence of patch embeddings $\left[z_{1}^{\left(0\right)},z_{2}^{\left(0\right)},\ldots ,z_{N}^{\left(0\right)}\right]$.

**Class Token and Positional Embeddings:** Following the standard ViT approach, we prepend a learnable class token $z_{\mathrm{class}}\in R^{D}$ to the sequence $Z^{\left(0\right)}=\left[z_{\mathrm{class}}^{\left(0\right)},z_{1}^{\left(0\right)},z_{2}^{\left(0\right)},\ldots ,z_{N}^{\left(0\right)}\right]$. This class token serves as a dedicated feature that aggregates information from all patches via the transformer layers and ultimately is used for classification. To retain the ordering and position of each patch in both space and time, we add a learnable positional embedding $E_{\mathrm{pos}}\in R^{(N+1)\times D}$. Unlike textual transformers, where positional encodings often follow a sine/cosine pattern, we use learnable embeddings. Each 3D patch position (defined by its temporal segment and spatial block indices) is assigned a unique embedding vector. The final input to the transformer is $Z^{\left(0\right)}\leftarrow Z^{\left(0\right)}+E_{\mathrm{pos}}$.

**Transformer Encoder:** The transformer encoder is composed of *L* identical layers, each containing a Multi-Head Self-Attention (MHSA) block followed by a position-wise Feed-Forward Network (FFN). Each layer uses residual connections and layer normalization to stabilize training and enable deep architectures.

Multi-Head Self-Attention (MHSA): For an input sequence $Z\in R^{(N+1)\times D}$, we compute queries *Q*, keys *K*, and values *V* as $Q=ZW^{Q},\;K=ZW^{K},\;V=ZW^{V}$, with *h* attention heads, we split *D* into *h* sub-dimensions of size $D_{h}=D/h$. Each head computes attention as ${\mathrm{head}}_{i}\left(Z\right)=\mathrm{softmax}\left(\frac{Q_{i}K_{i}^{T}}{\sqrt{D_{h}}}\right)V_{i}$. The outputs of all heads are concatenated and projected back: $\mathrm{MHSA}\left(Z\right)=\left[{\mathrm{head}}_{1};\ldots ;{\mathrm{head}}_{h}\right]W^{O}$.

Feed-Forward Network (FFN): Each layer’s FFN consists of two linear transformations with a non-linear activation (e.g., ReLU) in between: $\mathrm{FFN}\left(Z\right)=\eta \left(ZW_{1}+b_{1}\right)W_{2}+b_{2}$.

**Classification Head:** Since this is a binary classification problem, we only need a single scalar output representing the probability of the positive class. We apply a linear projection from the class token embedding to a single logit $\hat{y}=\sigma \left(z_{\mathrm{class}}^{\left(L\right)}w_{\mathrm{cls}}+b_{\mathrm{cls}}\right)$, where $w_{\mathrm{cls}}\in R^{D}$, $b_{\mathrm{cls}}\in R$, and σ(·) is the sigmoid function. Here, $\hat{y}$ is a probability ∈[0,1].

**Figure 2:**
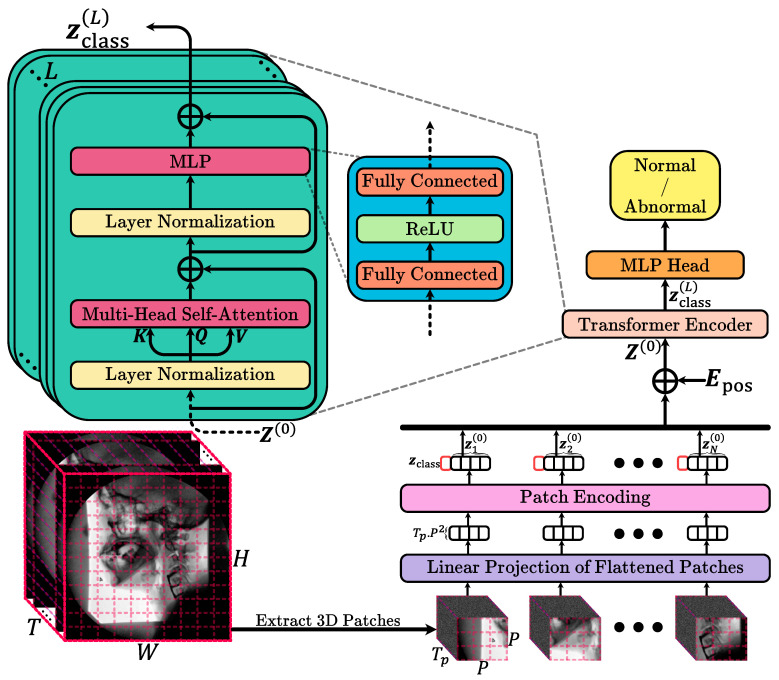
Diagram of the proposed algorithm, illustrating the processing pipeline including 3D patch extraction, embedding, transformer encoder, and classification head.

**Loss Function and Training:** We use binary cross-entropy (BCE) loss to train the model. The overview of the model is showed in Figure 2. All experiments were performed on an NVIDIA A100 GPU with 80 GB of RAM. The selection of hyperparameters was carried out via a grid search over a range of reasonable values. For memory efficiency, each video frame was isotropically resized to 256 by 256 pixels prior to spatial patching. The spatial patch size was set to 16 by 16, while the temporal patch size was set to 25 frames. The embedding dimension for the model was 64, and the MHSA mechanism employed 8 attention heads. In this configuration, the temporal window of 25 frames corresponds to approximately one second at 25 frames per second and was chosen so that each 3D patch is likely to contain a complete or nearly complete swallow, the 16 by 16 spatial patch size represents the smallest patch that still captures local oropharyngeal and laryngeal detail without making the token sequence prohibitively large, and the embedding dimension of 64 was selected from a small grid of candidate values as a compromise between validation performance, representational capacity, and computational cost. Eight transformer encoder layers were used, and the classification MLP head featured two layers with dimensions 128 and 64, respectively. The dataset was divided into training, validation, and testing sets at the subject level to prevent data leakage and artificial inflation of the model’s performance. In this approach, each subject’s entire data is exclusively assigned to a single group, with 10% (188 videos) reserved for testing and the remaining data split into training and validation sets at a ratio of 0.8:0.2. Data augmentation strategies, including rotations (within $\pm 5^{\circ }$), zoom (up to +10%), and the addition of Gaussian noise, were applied to the training set to avoid overfitting and to diversify the dataset. The model was trained for 200 epochs using the Adam optimizer with a learning rate of $10^{-3}$. On the available GPU hardware, training for 200 epochs required approximately 23 h, and inference on a single full length VFSS study took about 10 s.

## 5. Results

**Figure 3:**
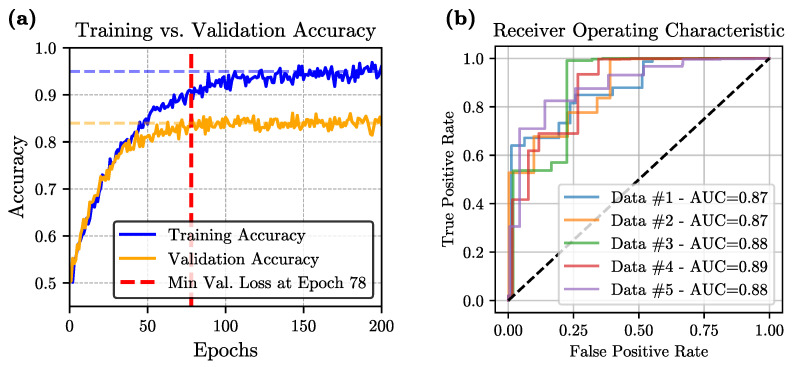
Performance outcomes of the proposed model: (**a**) Training and validation accuracy curves for one of the folds; (**b**) Receiver Operating Characteristic (ROC) curves derived from 5-fold cross-validation.

**Training and Validation Performance:** During training, we employed a 5-fold cross-validation strategy to mitigate overfitting and ensure robust performance. Across all folds, both training and validation losses initially decreased, indicating effective learning. However, as training progressed, an eventual increase in validation loss was observed in every fold, signaling the onset of overfitting. To address this, we adopted an early-stopping criterion based on the minimum observed validation loss, ensuring that the final model was selected at the point of best generalization rather than the end of the training process. Figure 3a illustrates the evolution of accuracy over the training epochs for one of the folds.

**Performance Metrics:** The final model, evaluated using 5-fold cross-validation, achieves an average accuracy of 84.37 ± 1.15%, sensitivity of 90.81 ± 2.11%, specificity of 79.49 ± 1.66%, precision of 82.94 ± 2.76%, and an F1-score of 85.68 ± 1.54%. The relatively high average accuracy demonstrates a strong overall predictive capability, while the model’s high sensitivity—reflected in a low rate of false negatives—underscores its potential reliability in clinical scenarios. The remaining metrics, including specificity, precision, and F1-score, also maintain consistently high values across folds, further confirming the model’s effectiveness. Taken together, these results highlight the suitability of the ViT-based architecture for capturing the spatio-temporal complexity intrinsic to the video data and generating reliable binary classification decisions.

**Figure 4:**
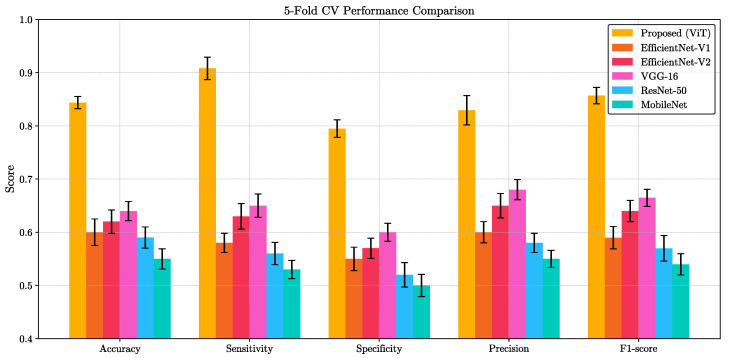
Five-fold cross-validation performance on VFSS abnormality classification for the proposed ViT and five CNN baselines (VGG-16, ResNet-50, EfficientNet-V1, EfficientNet-V2, MobileNet). Bars show mean scores for accuracy, sensitivity, specificity, precision, and F1-score; error bars denote ±1 SD across folds. The ViT attains the highest values on all metrics, with paired *t*-tests across folds confirming significant improvements over each baseline (*p* < 0.05).

**Statistical Analysis:** A comprehensive statistical evaluation was conducted to ensure the robustness of our model within the 5-fold cross-validation framework. The average area-under-the-curve (AUC) was 0.878, and the corresponding Receiver Operating Characteristic (ROC) curve is presented in Figure 3b. We also evaluated our ViT model against five widely-used convolutional architectures—VGG-16, ResNet-50, EfficientNet-V1, EfficientNet-V2, and MobileNet—on the same binary classification task. For each method, we report the mean and standard deviation of five key performance indicators (accuracy, sensitivity, specificity, precision, and F1-score) computed over a 5-fold cross-validation scheme. As summarized in Figure 4, the proposed ViT consistently achieved the highest scores across all metrics, significantly outperforming other methods. Paired *t*-test confirmed that these improvements are statistically significant (*p* < 0.05) for all metrics. This clear performance margin not only demonstrates the superior representational capacity of transformer-based architectures for VFSS analysis but also underscores the potential clinical utility of our ViT model for robust, high-fidelity classification in real-world settings.

**Figure 5:**
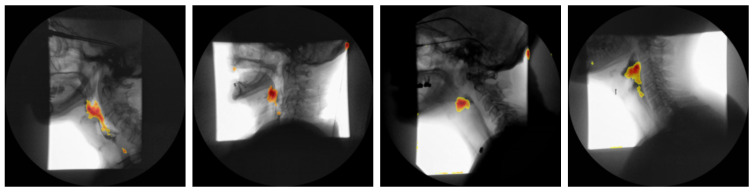
Attention-rollout saliency maps from the proposed ViT on four representative VFSS subjects overlaid on a “quiescent” frame (low optical flow). Warm colors consistently localize the oropharynx and hypopharynx, demonstrating precise, anatomically faithful localization.

**Attention Maps:** To elucidate the spatio-temporal evidence leveraged by our vision transformer classifier, we extracted the self attention matrices $A_{l}^{h}\in R^{N\times N}$ from every head *h* at each layer *l*. Following the relevance propagation scheme of Chefer et al. [27], the attention matrices were recursively multiplied to obtain a single relevance vector for the class token, which was then reshaped to the original P×P patch grid and bilinearly up sampled to full resolution. For every video frame *t*, the resulting saliency map $m_{t}\left(x,y\right)$ was normalized to [0,1] and weighted by the frame wise softmax confidence $\omega_{t}$. The cumulative contribution map $M\left(x,y\right)=\sum_{t=1}^{T}w_{t}\;m_{t}\left(x,y\right)$ was thresholded at the 90th percentile to generate a mask Ω. The mask was finally superimposed on a representative “quiescent” frame (mean optical flow < 0.5 px) to minimize motion artifacts and facilitate anatomical interpretation. Figure 5 presents the attention maps generated for four representative subjects.

## 6. Discussion

The ViT-based approach to binary VFSS classification demonstrates that leveraging 3D patches and attention mechanisms can effectively capture the spatio-temporal complexity inherent in video data. By representing input sequences as volumetric tokens, the model is able to learn long-range dependencies across both space and time, moving beyond the limitations of more traditional convolution-based methods.

Our work is driven primarily by clinical applicability while still incorporating state-of-the-art methodology. On the one hand, the transformer-based design and 3D patch tokenization address the spatio-temporal subtleties of VFSS, underscoring a forward-looking approach that harnesses cutting-edge deep learning techniques. The choice of a pure ViT approach is further justified by its scalability with massive datasets in clinical environments which enables the learning of rich, high-level representations that capture long-range dependencies more effectively than local convolutional filters. Moreover, the simpler and more uniform architectural design of a pure ViT obviates the need to integrate CNN modules for local feature extraction, thereby facilitating easier optimization, parallelization, and reduced training complexity on modern hardware. The self-attention mechanism inherent to ViTs not only improves interpretability—by providing visualizable attention maps that highlight key regions in the data that can be utilized by SLPs for diagnosis purposes—but also offers robustness to noise and perturbations, which is crucial given the variability in medical imaging quality. In addition, transformers naturally capture global context and can process entire sequences in parallel, enabling the detection of subtle anomalies that may emerge over extended periods or across disparate spatial regions. This approach also reduces reliance on convolutional inductive biases, allowing the model to learn custom feature representations directly from data, and scales gracefully with increases in both model size and dataset volume. Complementing these design choices, the use of learned positional embeddings, attention-based redundancy handling, and a single unified framework for spatio-temporal processing simplifies the overall model architecture by avoiding the complexity of hybrid CNN-ViT designs, ultimately enhancing both performance and ease of deployment. The framework is also intrinsically amenable to multimodal integration—textual or clinical notes can be fused into the pure ViT with minimal modification—positioning this method at the cutting edge of clinical data analysis. Operationally, the fully automated VFSS preprocessing pipeline processes data without manual frame selection or other human interventions, making it practical in real-world clinical settings. By minimizing user input and enabling consistent processing, the system operates with greater efficiency and objectivity, ultimately facilitating broader adoption.

One key finding from the training process was that overfitting began to manifest during the early epochs of the 5-fold cross-validation, as indicated by an increase in the validation loss. Although this behavior might indicate limited generalization, the use of early stopping based on the minimum validation loss proved instrumental in mitigating these concerns. As a result, the final outcomes highlight its proficiency in both accuracy and class-specific metrics. High sensitivity, for instance, is particularly beneficial in clinical scenarios where missing an abnormal event could be critical. This robust sensitivity, combined with moderate specificity and precision, suggests that the approach may support human decision-making. Moreover, the strong F1-score demonstrates a consistent balance between precision and recall, further underscoring the model’s reliability. The ROC curve in Figure 3b, together with the mean AUC of 0.878, indicates that the model achieves good discrimination between normal and abnormal studies, and the 95% confidence intervals reported for accuracy, sensitivity, specificity, F1-score, and AUC across the five folds suggest that these performance estimates are reasonably stable across different train-test partitions, while still reflecting the uncertainty inherent in a single center dataset and underscoring the importance of future validation on external cohorts.

It is also worth noting that the applied architectural choices, such as the use of a relatively small embedding dimension and a moderate number of transformer encoder layers, allowed for manageable complexity while still achieving strong results. Moreover, selecting a subset of frames for classification and using large spatial and temporal patches enabled the model to focus on clinically or contextually relevant segments of the video without being overwhelmed by excessive temporal or spatial detail.

In terms of the accuracy–complexity trade off, Table 2 shows that the proposed ViT has the largest computational footprint, with approximately 196 GFLOPS and 121 million parameters, compared with substantially lighter CNN baselines. Nonetheless, across the 5-fold cross validation the ViT achieved the highest accuracy, sensitivity, specificity, precision, F1-score, and AUC, and these gains were statistically significant, indicating that the additional capacity is effectively used rather than simply increasing model size. At the same time, inference remains practical on contemporary clinical or research GPUs, with end-to-end prediction for a full VFSS study requiring only several seconds, so the higher complexity is unlikely to be a limiting factor for deployment in routine workflows.

As shown in Figure 5, the attention-rollout maps produced by our transformer model consistently demarcate two key anatomical regions: the oropharynx and the hypopharynx. These areas are among the key regions SLPs scrutinize when assessing for penetration, aspiration, or post-swallow residue during VFSS. Notably, earlier study employing Grad-CAM in convolutional neural networks designed for airway-invasion detection has reported analogous localization patterns [28]. However, our findings indicate that transformer-based attention rollout not only recovers this clinically relevant region but does so with markedly higher spatial precision and contrast, yielding sharper, more anatomically accurate saliency maps. This improvement in fidelity aligns with recent advances in transformer explainability, which demonstrate that multi-headed self-attention mechanisms can capture long-range dependencies and fine-grained spatial cues more effectively than convolutional counterparts. Consequently, the present work underscores the potential of transformer architectures to enhance interpretability in medical imaging applications, providing saliency visualizations that are both biologically meaningful and diagnostically actionable.

Although VFSS interpretation by SLPs inevitably involves some degree of subjectivity, particularly when assigning detailed PAS, reliability is generally higher for coarser distinctions than for exact PAS levels. In this study, we therefore framed the task as a binary classification of studies into normal versus abnormal swallows, which is less granular and expected to yield more consistent labels across raters than multi-level PAS ratings, even though some inter-rater variability will remain. This residual subjectivity effectively sets an upper bound on achievable model performance and may partly determine which cases are counted as false positives or false negatives. As such, future work should explicitly quantify inter-rater agreement for the binary labels and explore the use of multi-rater or consensus annotations to further stabilize the training signal and better characterize the relationship between annotation variability and model behavior.

In interpreting these findings, it is important to recognize that the current model uses a global binary normal versus abnormal label at the study level, which intentionally aggregates a wide spectrum of pathophysiology and severity, from mild residue to frank aspiration, and therefore limits the immediate prognostic and therapeutic specificity of its outputs. This design reflects the primary goal of the present work, which is to provide a fully automated proof-of-concept that VFSS can be classified in a weakly-supervised manner from whole sequences without manual frame selection or region of interest annotation, rather than to deliver a fully mature clinical decision-support system. Because abnormality type and severity are not available in a structured and consistent way in our dataset, and many specific patterns occur only infrequently, reliable subgroup analyses across distinct abnormal categories are not yet feasible and would risk being statistically underpowered and potentially misleading. A similar constraint applies to bolus characteristics, since bolus consistency is not encoded in a standardized fashion at the level of individual swallows, meaning that the present model is not designed to offer bolus specific or diet specific recommendations. In addition, patients with pronounced movement disorders such as chorea or cervical dystonia are likely underrepresented in the current dataset, and although the model has been trained and evaluated on VFSS data that include typical degrees of patient repositioning, modest voluntary and involuntary motion, and minor scanner related artifacts, its performance in cohorts with more severe and irregular head and neck movement remains uncertain. Future work will therefore require prospectively collected VFSS datasets with detailed and standardized annotations of abnormality type and severity, carefully recorded bolus consistency information, and enriched representation of patients with significant movement disorders, so that the present weakly-supervised framework can be extended to multiclass or multilabel outputs, rigorously evaluated in clinically important subgroups, and ultimately aligned more directly with abnormality specific prognosis, dietary modification, and tailored intervention planning.

Taken together, these limitations and opportunities point toward future extensions that enrich the model’s predictive capabilities while maintaining a strong emphasis on transparency, fairness, and clinical usability. Beyond the development of prospectively collected, richly annotated VFSS cohorts, it will be important to evaluate the framework on multi-site datasets acquired with different imaging devices to better characterize domain shift and improve generalizability, while implementing more rigorous, multi-rater annotation protocols to mitigate labeling bias. From a methodological standpoint, further refinement of spatio-temporal positional encoding, coupled with domain adaptation strategies and transfer learning from large-scale video datasets, may help sustain performance when annotated VFSS data remain limited. Finally, parallel work on interpretable visualization tools and clinician-centered interfaces will be essential to ensure that the model’s outputs can be understood, trusted, and effectively integrated into routine dysphagia assessment and management.

## 7. Conclusions

This study introduced a ViT-based model for binary video classification using 3D patches to capture the spatio-temporal dependencies of VFSS. Early stopping based on the minimum validation loss helped mitigate overfitting, resulting in strong performance across multiple metrics. High sensitivity, balanced by robust specificity, precision, and F1-score, underscores the model’s potential utility in critical settings where accurate detection of abnormalities is crucial.

Nonetheless, further work is needed. Larger, more diverse datasets and multi-site data could enhance generalizability and reduce labeling biases. Improving model interpretability and tailoring its outputs to the needs of domain experts remain important goals. Finally, expanding the classification scheme beyond binary categories may enable more detailed and clinically relevant insights.

## Figures and Tables

**Table 1:** Scanner-wise distribution of VFSS clips and clip length characteristics.

Scanner Vendor	Total VFSS Clips, *n*	Eligible VFSS Clips After Exclusion, *n*	Clip Length, Mean ± SD (Frames)
Kodak CR950	1	1	112 ± 0
NAI Tech Products MDR Video	199	70	105.1 ± 19.3
PACSGEAR MDR Video	1727	765	102.8 ± 23.5
Siemens Fluorospot Compact	787	318	103.6 ± 20.1
**All scanners**	**2714**	**1154**	**103.4 ± 22.4**

**Table 2:** Comparison of computational cost and model size for the proposed ViT and baseline CNN architectures.

Model	GFLOPS	No. of Parameters
MobileNet	1.2	3 M
EfficientNet-V1	1.0	6 M
EfficientNet-V2	1.6	7 M
ResNet-50	4.5	25 M
VGG-16	35	40 M
**Proposed**	**196**	**121 M**

## Data Availability

The datasets presented in this article are not readily available because privacy and ethical restrictions. Requests to access the datasets should be directed to the corresponding author.

## Abbreviations

The following abbreviations are used in this manuscript:

VFSS	Videofluoroscopic Swallowing Study
ViT	Vision Transformer
CNN	Convolutional Neural Network
AUC	Area-Under-The-Curve
SLP	Speech-Language Pathologist
CLAHE	Contrast-Limited Adaptive Histogram Equalization
MHSA	Multi-Head Self-Attention
FFN	Feed-Forward Network
MLP	Multi-Layer Perceptron
BCE	Binary Cross-Entropy
CV	Cross-Validation
ROC	Receiver Operating Characteristic
